# Motor and sensory disturbances induced by sensorimotor conflicts during passive and active movements in healthy participants

**DOI:** 10.1371/journal.pone.0203206

**Published:** 2018-08-29

**Authors:** Clémentine Brun, Martin Gagné, Candida S. McCabe, Catherine Mercier

**Affiliations:** 1 Center for Interdisciplinary Research in Rehabilitation and Social Integration (CIRRIS), Québec, QC, Canada; 2 Department of Rehabilitation, Laval University, Québec, QC, Canada; 3 Royal United Hospitals NHS Foundation Trust, Bath, United Kingdom; 4 University of the West of England, Bristol, United Kingdom; 5 The Florence Nightingale Foundation, London, United Kingdom; University of Ottawa, CANADA

## Abstract

Sensorimotor conflict induces both sensory and motor disturbances, but the specific factors playing a role in conflict-induced disturbances are still misunderstood. For example, we still do not know the role played by motor intention (vs. a purely visuo-proprioceptive conflict) or the influence of specific types of incongruent visual feedback. The objective of this study was threefold: 1- to compare the effect of passive and active movement during sensorimotor conflict on sensory disturbances measured with a questionnaire; 2- to compare the effect of three incongruent visual feedback conditions on sensory and motor (mediolateral drift and movement amplitude) disturbances; 3- to test whether conflict-induced sensory and motor disturbances were stable over time. 20 healthy participants realized active or passive cyclic upper limb movements while viewing either congruent or incongruent visual feedback about their movement using a robotized exoskeleton combined with 2D virtual reality interface. First, results showed that in condition of conflict, participants reported higher sensory disturbances during active movements compared to passive movements (p = 0.034), suggesting that the efference copy reinforces the conflict between vision and proprioception. Second, the three conditions of incongruence in the active condition induced similar sensory (all p>0.45) and motor disturbances (medio-lateral drift: all p>0.59 and amplitude: all p>0.25), suggesting that conflict induced motor disturbances could be related more to the observation of another movement rather than to a detection of conflict between motor intention and sensory feedback. Finally, both sensory and motor disturbances were stable over time (all ICCs between 0.76 and 0.87), demonstrating low variability within participants. Overall, our results suggest that the efference copy is more involved in sensory disturbances than in motor disturbances, suggesting that they might rely on independent processes.

## Introduction

In order to produce accurate and adapted movements, proprioceptive and visual information about limb movements are systematically integrated [1] and compared to the predicted sensory feedback arising from our motor intentions [2,3]. Generally, we are unaware of the actual state of the system, except when discrepancies occur [2]. Sensorimotor conflict, defined as a mismatch between the predicted and the actual sensory feedback, produces both sensory and motor disturbances [4]. Sensorimotor conflict can be elicited easily using a mirror, for example by asking participants to perform active out-of-phase bilateral arm movements while viewing a mirror positioned in the sagittal plane, thus giving the illusion of in-phase bilateral arm movements [5,6]. Studying sensorimotor conflicts provides a better understanding of how the brain integrates sensory and motor information, and how it interprets (sensory disturbances) and responds (motor disturbances) to such conflicts. However, using a mirror to elicit a conflict presents some limitations. First, both upper (or lower) limbs need to be used, while interlimb coupling can impact on both sensory integration [7] and motor behaviour [8,9] in the mirror box paradigm. Second, the mirror paradigm does not allow to study various conditions of conflicts. Interestingly, recent developments in virtual reality and robotics provide much more flexible ways of inducing a conflict, allowing us to assess in a more specific manner the factors playing a role in conflict-induced disturbances.

Sensory disturbances induced by sensorimotor conflicts have been mainly studied in the field of pain research. It has been shown in various chronic pain conditions that sensorimotor conflict transiently induces sensory disturbances, such as changes in pain perception, feelings of peculiarity, changes in perceived weight and in temperature of the limb, and the impression of losing or gaining a limb (for a systematic review see Don and collaborators [10]). In healthy participants, sensorimotor conflict also induces sensory disturbances, although to a lesser extent than chronic pain patients [4,5,11–13]). Although all these studies refer to sensorimotor conflict, one important aspect is that they all focused on active movement, and therefore it is not possible to determine whether the observed sensory disturbances result from a conflict between vision and proprioception (visuo-proprioceptive conflict) or between vision and motor intention (visuo-motor conflict). To address that question, the first objective of this study was to compare the sensory disturbances evoked by incongruent visual feedback about movement (relative to congruent feedback) depending on whether the movement was passive (visuo-proprioceptive conflict) or active (visuo-motor conflict).

Sensorimotor conflicts are known to evoke motor disturbances in parallel to sensory disturbances [4]. Conflict-induced motor disturbances have been mainly studied with a mirror positioned in the frontal plane while participants were required to draw geometric shapes [14,15], or in the sagittal plane during reaching movements of both arms [16,17]. When the mirror is positioned in the frontal plane, participants have access to visual feedback about movement errors and are therefore able to do online corrections [14,15]. In contrast, in the studies with the mirror in a sagittal position, participants have no feedback about their movement as they are only viewing movement of the contralateral upper limb [16,17]. Altogether these studies raise the question whether having visual feedback about movement errors allows participants to modify online their performance. Attempt to correct movements online in the presence of incongruent feedback could potentially increase the conflict, and thus their motor and sensory disturbances. Thus, the second objective of the study was to compare the effect of three conditions of incongruent visual feedback (relative to congruent feedback) on motor disturbances, as well as on sensory disturbances.

Finally, a third objective, focussing more on methodological aspects, was to test whether sensory and motor disturbances induced by sensorimotor conflicts are stable over time, given that this type of measure is being used increasingly in the scientific literature [4–6,11,18–22].

## Materials and methods

### Participants and ethics statement

Twenty healthy volunteers (18 right-handed, 12 females, mean ± standard deviation (SD) age: 26.8±7.4 years) were recruited from Laval University. None of them had a self-reported history of visual, nervous system or musculoskeletal disease that could affect task performance. All participants provided their written informed consent prior to admission to the study. The experiment was performed in accordance with the tenets of the Declaration of Helsinki and the study protocol was approved by the local ethical review board (Institut de réadaptation en déficience physique de Québec, Canada, n°2015–461).

### Study design

Each participant took part in two experimental sessions separated by one week. In each session, the participant was exposed to six experimental conditions (see section 2.5 for more details): Active_Congruent, Active_Incongruent__XY_, Active_Incongruent__Y_, Active_Incongruent__Video_, Passive_Congruent, Passive_Incongruent__Video_ (2 trials by experimental condition, in a pseudorandom order, yielding a total of 12 trials per session).

### Instrumentation

**Fig 1:**
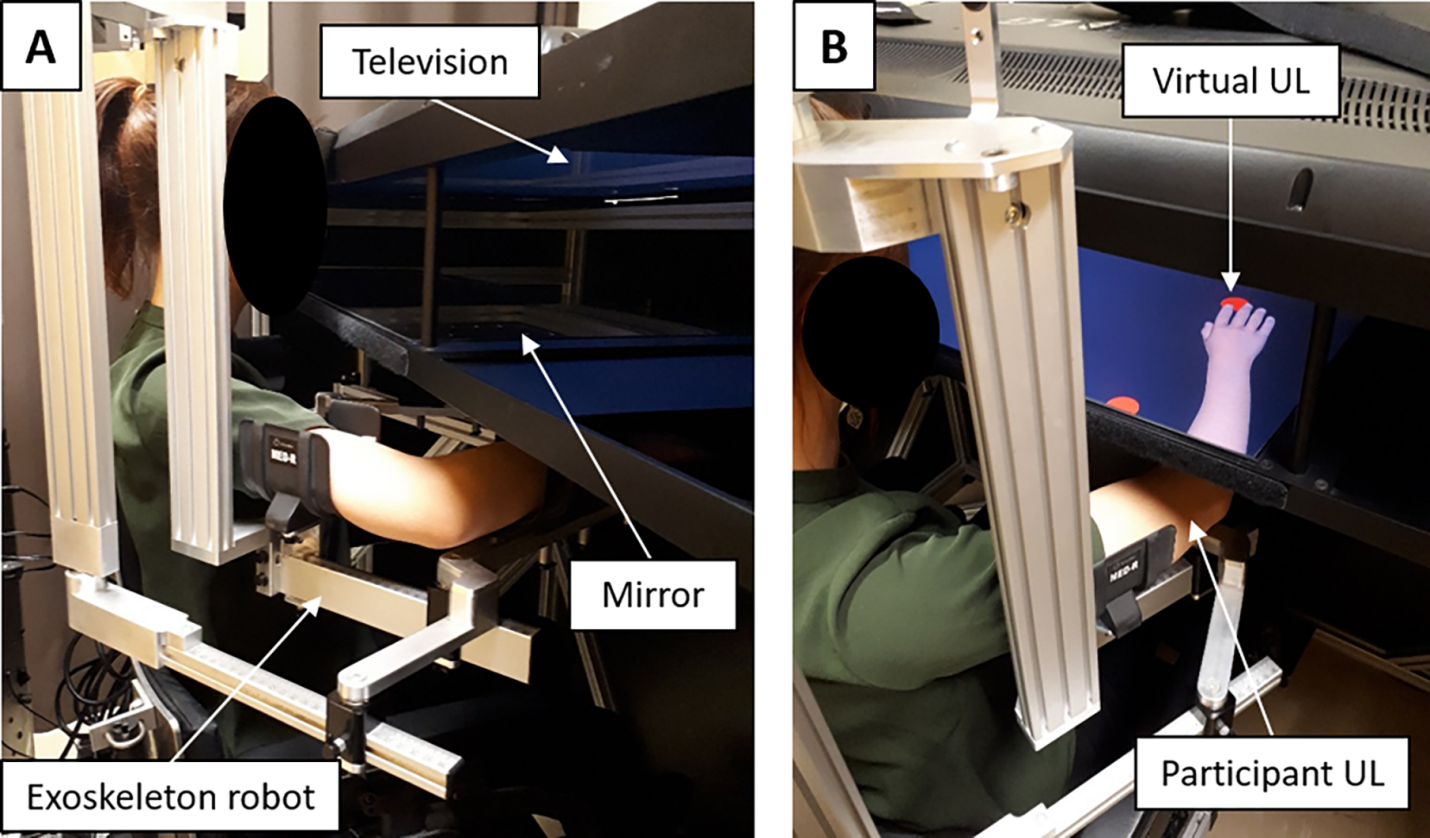
Experimental set up. The exoskeleton robot and 2D virtual environment are the 2 elements of the KINARM. (A) The exoskeleton is fitted to the anthropometric characteristics of the participant’s upper limb (UL). (B) The virtual environment consists in the projection of a virtual upper limb on a semi-transparent mirror (47”) thanks to a television. The UL rest on the exoskeleton under the semi-transparent mirror and is obstructed from the participant’s view.

The experimental task was conducted using a KINARM robotized exoskeleton (BKIN Technologies, Kingston ON, Canada; see Fig 1A), that allows shoulder abduction-adduction and elbow flexion-extension in order to move the upper limb (UL) in the horizontal plane (the weight of the UL being fully supported). The movement of the tested UL (dominant limb) was either active or passive, i.e. moved by the robot. The robot is interfaced with a 2D virtual environment (47 inches) creating the illusion of a virtual UL at the same location as the participant’s UL (Dexterit-E software version 3.4.2; Fig 1B), while the participant’s UL is obstructed from view. According to the visual feedback (VF) condition, the virtual UL was either driven in real-time by the participant’s actual movement or followed a pre-defined trajectory (see below). Joint angular positions for both the shoulder and elbow were obtained from KINARM motor encoders and sampled at 1 kHz, and the position of the index fingertip was computed in real-time. Data processing was made with Matlab (MathWorks, R2011b).

### Experimental task

The first session began with two familiarization trials (one Passive and one Active), in which the movement of the virtual UL reproduced faithfully the actual movement of the participant’s UL. Participants then moved on to the main experimental task.

**Fig 2:**
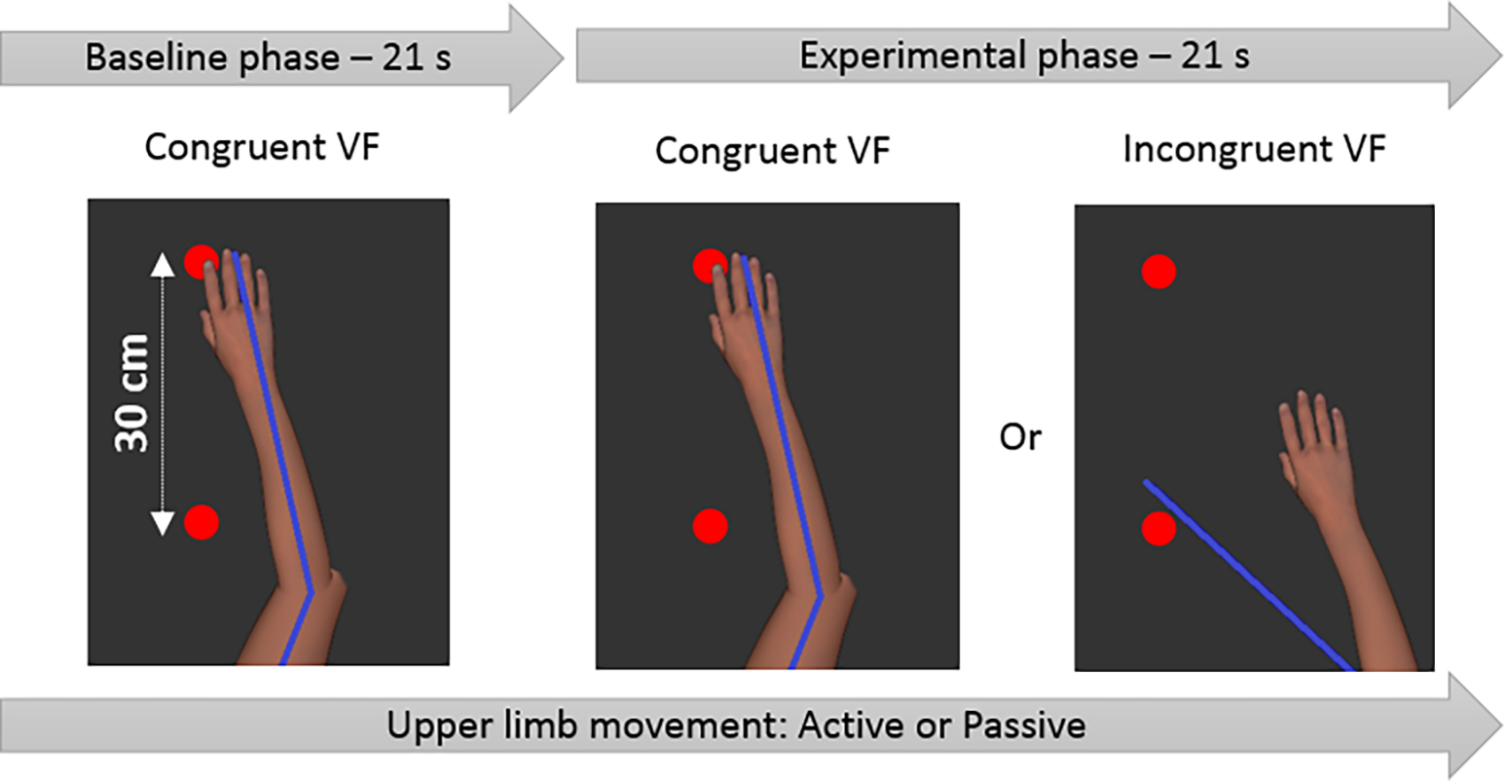
Timeline and conditions of visual feedback (VF). Participants saw exclusively the virtual upper limb and the red targets. Blue line depicts the real position of the upper limb. The movement of the upper limb could be either active of passive during all the trial (Baseline and Experimental phases). During the Active condition, participants were required to reach one of the targets at each metronome beat (0.33 Hz) in order to create a cyclic movement. In the Passive condition, the same movement frequency was created by the robot. In the Baseline phase, the virtual upper limb movement was always congruent with the actual participant movement. In the Experimental phase, the movement of the virtual upper limb was either congruent or incongruent depending on the experimental condition.

Before each trial, participants were informed of the Movement condition (Active or Passive), and then positioned their UL on a red target (or the robot passively moved the UL to the target) corresponding to an angular position of 40° for the elbow and 85° for the shoulder (initial position). The trial then comprised two phases (Fig 2).

In the Baseline phase (21 seconds), the virtual UL reproduced faithfully the movement of the participant’s UL. Two red targets appeared, one at (0, 15) coordinates (in cm) and the other at (0, -15) from the initial position, and remained until the end of the trial. Participants were required to successively reach each target without stopping on them, in order to create a cyclic movement as fluid and straight-lined as possible. A metronome beat was provided to help the participant maintain the required movement frequency (0.33 Hz).

In the Experimental phase (21 seconds), one of the congruent or incongruent VF conditions was presented to the participant. When the movement was active and during the incongruent VF conditions, participants were required to continue to reach each target as in the Baseline phase, even if the VF was disturbing.

After each trial, participants had to respond to a questionnaire about their perception of their UL (for more details, see section 2.6.)

### Experimental conditions

Six experimental conditions were used, in which the movement of the UL was either Passive or Active. In the Passive condition, participants were required to relax their muscles and not to follow or resist the movement of the robot. In the Active condition, participants had to perform the movement.

1. *Active_Congruent*: The virtual UL reproduced faithfully the participant’s UL movement. In that condition, the participant had direct control over the movement of the virtual UL, and then had access to visual feedback about his performance.
2. *Active_Incongruent_*_*XY*_: The (X,Y) coordinates of the index of the virtual UL were rotated by 90 degrees compared to the actual position of the index of the participant. In that condition, the participant had direct control over the movement of the virtual UL, and then had access to visual feedback about his performance.
3. *Active*_*Incongruent_*_*Y*_: The movement of the virtual UL was pre-programmed to move in the mediolateral axis (while the participant performed a movement in the anteroposterior axis), but the velocity and the movement amplitude of the virtual UL were driven by the participant’s actual movement, on the basis of the Y-axis coordinates of the index. In that condition, the participant had some control over the movement of the virtual UL (temporal, but not spatial match), and then had limited access to visual feedback about his performance.
4. *Active*_*Incongruent_*_*Video*_: The movement of the virtual UL was pre-programmed in the mediolateral axis, with a fixed amplitude of 30 cm. In that condition, the participant had no control over the movement of the virtual UL, and then no visual feedback about his performance.
5. *Passive_Congruent*: The virtual UL reproduced faithfully the participant UL movement.
6. *Passive_Incongruent_*_*Video*_: The movement of the virtual UL was pre-programmed in the mediolateral axis, with a fixed amplitude of 30 cm. This condition is equivalent to the Incongruent__XY_ and Incongruent__Y_ condition during passive movement. Indeed, the trajectory between the two red targets is always perfectly rectilinear and synchronized with the metronome beat as the movement is controlled by the robot.

#### Objective 1—Comparison of Active and Passive movement

In order to compare the sensory disturbances evoked by incongruent visual feedback about movement (relative to congruent feedback) depending on whether the movement was passive or active, the following conditions were analysed: Active_Congruent, Passive_Congruent, Active_Incongruent__Video_ and Passive_Incongruent__Video_. Only one incongruent active condition was used for that objective, and the Active_Incongruent__Video_ was selected to provide similar visual feedback in both the Active and Passive conditions.

#### Objective 2—Comparisons between three conditions of incongruence

In order to compare the effect of various conditions of visual feedback on motor and sensory disturbances, the four visual feedback conditions during the active movement were used for that objective: Active_Congruent, Active_Incongruent__XY_, Active_Incongruent__Y_ and Active_Incongruent__Video_.

#### Objective 3—Test-retest reliability

In order to assess the test-retest reliability on motor and sensory disturbances the three conditions of sensorimotor conflict during the Active condition were used: Active_Incongruent__XY_ or Active_Incongruent__Y_ or Active_Incongruent__Video_.

### Measures and data analyses

#### Sensory disturbances

At the end of each trial, participants verbally answered eight questions assessing changes in UL perception including: pain, discomfort, a perceived lost limb, temperature change, weight change, a perceived extra-limb, losing control and feelings of peculiarity [5,6,19]. Table 1 describes the questions in English and in French. Participants were required to rate changes from the Baseline phase on a scale from 0 to 3 (0 = no change; 1 = low change; 2 = moderate change; 3 = high change). A total score was computed using the mean of the eight items. In the literature, it has been shown that healthy participants report on average three sensory disturbance items, mainly characterized by the impression of gaining a limb, feelings of peculiarity and losing control [4,19,23]. In our experiment, a total score of 0.25 means that participants reported a score of 1 (= low change) for two items or a score of 2 (= moderate change) for one item. Therefore, a total score inferior to 0.25 was considered to be small and a total score superior to 0.0.25 was considered to be important.

**Table 1:** Questionnaire of sensory disturbances.

English version	French version
0 = no change	0 = aucun changement
1 = low change	1 = changement faible
2 = moderate change	2 = changement modéré
3 = high change	3 = changement important
1. Did you perceive any changes in painful sensation?	1. Avez-vous ressenti un changement au niveau de la sensation de douleur?
2. Did you perceive any changes in discomfort sensation?	2. Avez-vous ressenti un changement au niveau de la sensation désagréable?
3. Did you have the feeling of losing your limb?	3. Avez-vous eu l’impression de perdre votre bras?
4. Did you feel a change in your upper limb temperature, as your limb getting colder or hotter?	4. Avez-vous ressenti un changement au niveau de la température de votre bras, comme s’il devenait plus chaud ou plus froid?
5. Did you feel a change in your upper limb weight, as your limb getting heavier or lighter?	5. Avez-vous ressenti un changement au niveau du poids de votre bras, comme s’il devenait plus lourd ou plus léger?
6. Did you have the feeling of gaining a limb?	6. Avez-vous eu l’impression d’avoir un bras supplémentaire?
7. Did you have the feeling of losing control?	7. Avez-vous eu l’impression de perdre le contrôle?
8. Did you feel any strange or peculiar sensations?	8. Avez-vous ressenti des sensations étranges / bizarres?

For each question, participants were required to rate changes from the baseline phase on a scale from 0 to 3.

#### Motor disturbances

Two outcomes were used to assess motor disturbances [4] based on the position of the index fingertip:

- Amplitude: y-coordinates were encoded for each peak of flexion and extension. For each movement half-cycle, the amplitude on the y-axis was extracted.
- Medio-lateral drift: for each movement half-cycle, the x-coordinate of the maximal deviant point was extracted. A negative value indicates a medial drift and a positive value a lateral drift.

### Statistics

The mean±SD are reported in the results. The threshold for statistical significance was set to p<0.05. Normality of the data has been assessed with the Komolgorov Smirnov test. For the sensory disturbances, one participant was excluded because his score was superior to 4 SD from the mean.

#### Objective 1—Comparison of Active and Passive movement

A 2 x 2 repeated measures analysis of variance (rmANOVA) was used: [*Movement* (Active or Passive) x *Visual Feedback* (Congruent or Incongruent)] was performed. Post hoc tests were performed using Tukey corrections.

#### Objective 2—Comparisons between three conditions of incongruence

The four visual feedback conditions during the active movement were analysed using a one-way ANOVA in a within subject design: *Visual Feedback* (Active_Congruent or Active_Incongruent__XY_ or Active_Incongruent__Y_ or Active_Incongruent__Video_). For each outcomes, p-values were Greenhouse-Geisser corrected for sphericity. Post-hoc tests were performed using REGWQ (Ryan/Einot and Gabriel/Welsh test) correction for multiple comparisons, a procedure that is more powerful than Tukey for one-way ANOVA [24]. Note that passive conditions were not used for that objective, given that no motor disturbances can be measured in that condition.

#### Objective 3—Test-retest reliability

Test-retest reliability of conflict-evoked sensory and motor disturbances over time was assessed using intraclass correlation coefficient (ICC) for the incongruent VF conditions during active movement. ICC [95% confidence interval] are reported in the results. A minimum of 0.70 is recommended to ensure us that a measure is reliable [25].

## Results

The majority of the participants reported changes in peculiarity (55%), the perception to having an extra limb (55%) and the impression of losing control (70%) in at least one experimental condition. Some participants reported discomfort (30%), a lost limb perception (30%), changes in weight and temperature (respectively 40% and 30%), and one (5%) participant reported painful sensations.

### Objective 1—Comparison of Active and Passive movement

**Fig 3:**
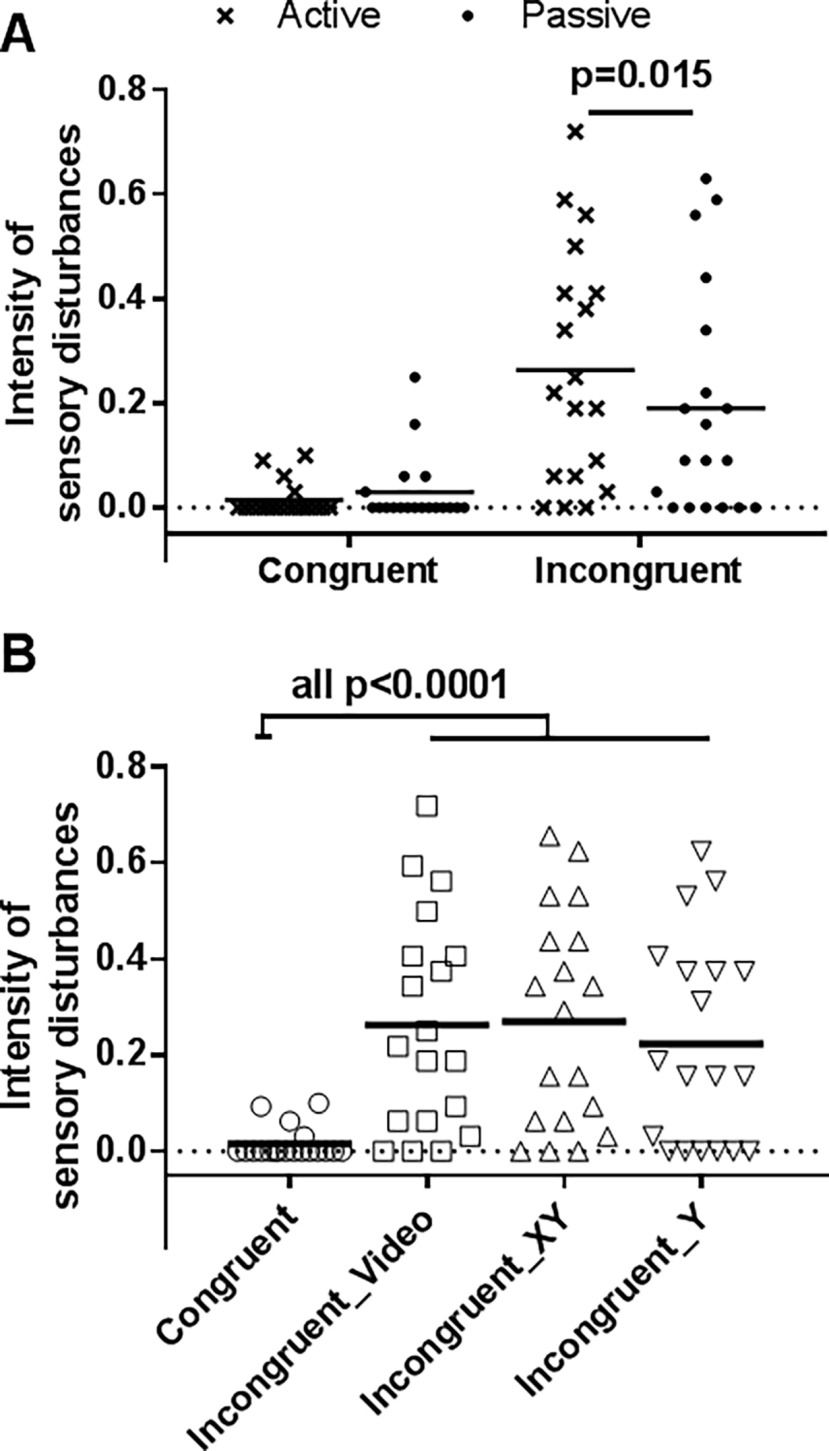
Intensity of sensory disturbances. (A) Objective 1: the two conditions of visual feedback (Congruent or Incongruent) during the Passive and Active movement conditions. (B) Objective 2: the four visual conditions during the active movement condition. Errors bars represent the standard error of the mean.

As expected, a significant main effect of Visual Feedback (F(1,18) = 21; p<0.001; ɳ_p_ = 0.53) was found, participants reporting higher sensory disturbances in condition of incongruence (Congruent = 0.022±0.039, Incongruent = 0.23±0.20, mean difference [95% confidence intervals (CI)] = 0.21 [0.13 to 0.29]; see Fig 3A). No significant main effect of Movement was observed (F(1,18) = 1.5; p = 0.24), but a significant Visual Feedback X Movement interaction effect was observed (F(1,18) = 5.3; p = 0.034; ɳ_p_ = 0.22). The interaction revealed that Active movement significantly induced higher sensory disturbances than Passive movement only in the Incongruent VF condition. Indeed, post hoc analyses revealed a significant difference between the Active and Passive condition during Incongruent VF (mean difference [95%CI] = 0.076 [0.004 to 0.072], p = 0.015, d = 0.41) but not during the Congruent VF condition (mean difference [95%CI] = -0.014 [-0.017 to -0.011], p = 0.59, d = 0.21).

### Objective 2—Comparisons between three conditions of incongruence

#### Sensory disturbances

A significant main effect of Visual Feedback was observed (F(3,54) = 13.3; p<0.001, ɳ_p_ = 0.52). As shown on Fig 3B participants reported more sensory disturbances in all the three conditions of incongruence compared to the Congruent condition (Congruent vs Incongruent__video_: mean difference [95%CI] = 0.24 [0.15 to 0.33], p<0.001; Congruent vs Incongruent__XY_: mean difference [95%CI] = 0.25 [0.16 to 0.34], p<0.001; Congruent vs Incongruent__Y_: mean difference [95%CI] = 0.21 [0.12 to 0.30], p<0.001). However, the three sensorimotor conflicts conditions did not differ from each other (Incongruent__video_ vs Incongruent__XY_: mean difference [95%CI] = 0.007 [-0.033 to 0.047], p = 0.97; Incongruent__video_ vs Incongruent__Y_: mean difference [95%CI] = -0.04 [-0.08 to 0], p = 0.52; Incongruent__XY_ vs Incongruent__Y_: mean difference [95%CI] = -0.04 [-0.08 to 0], p = 0.45).

#### Motor disturbances

**Fig 4:**
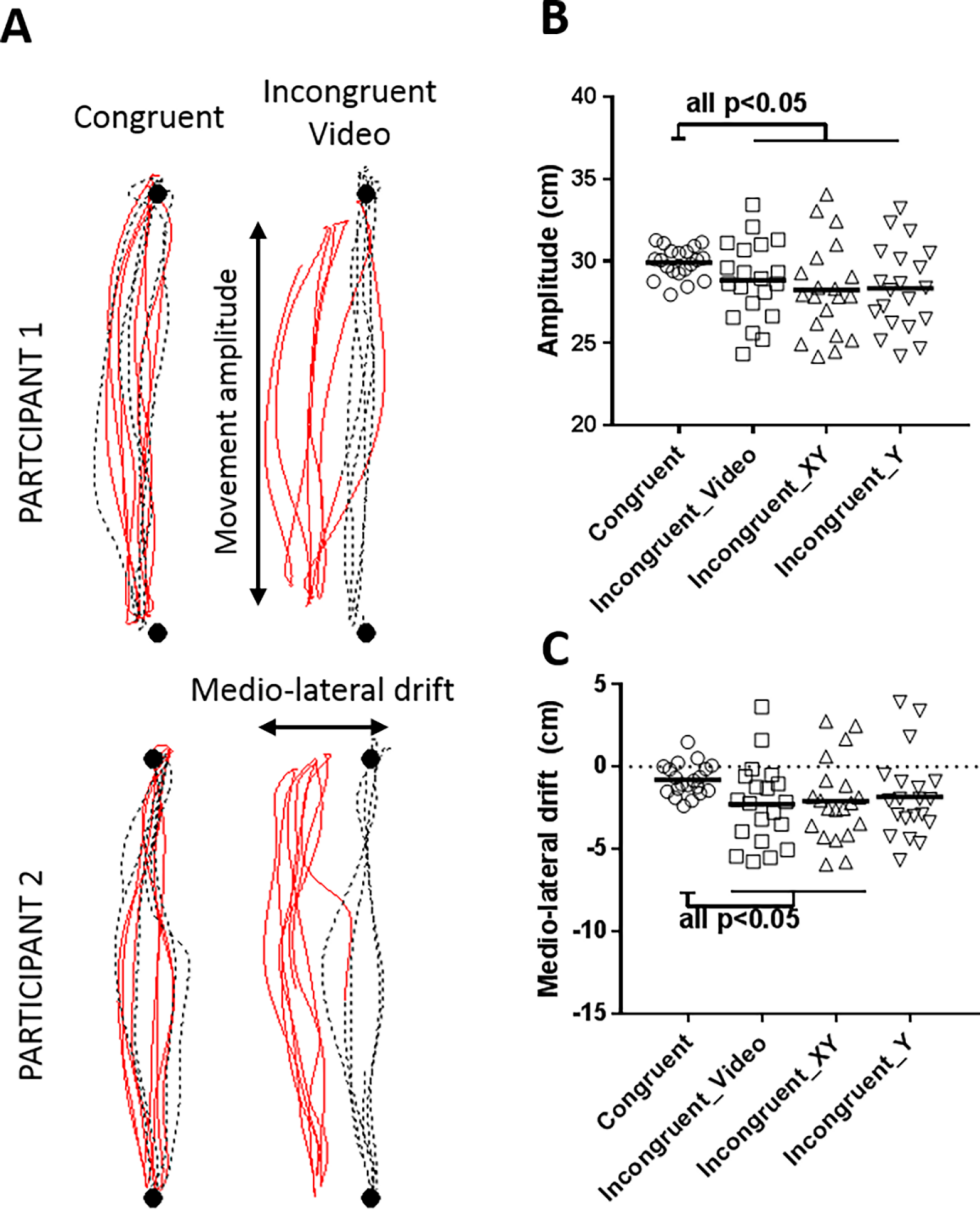
Motor disturbances (Objective 2). (A) Individual data for two representative participants. Black circles, dashed black lines and full red lines represent, respectively, targets, trajectory of the fingertip during the baseline phase and trajectory of the fingertip during the Experimental phase. (B) Movement amplitude during the Experimental phase. C: Medio-lateral drift during the Experimental phase. A negative value indicates a medial drift. Error bars represent the standard error of the mean.

Fig 4A provides an example of motor disturbances induced by sensorimotor conflicts.

Amplitude: A significant main effect of Visual Feedback was observed (F(3,57) = 8.81; p = 0.008, ɳ_p_ = 0.32). As shown on Fig 4B, in all conditions of incongruence the movement amplitude was significantly lower compared to Congruent VF condition (Congruent vs Incongruent__video_: mean difference [95%CI] = -1.2 [-2 to -0.04], p = 0.008; Congruent vs Incongruent__XY_: mean difference [95%CI] = -1.8 [-2.8 to -0.8], p<0.001; Congruent vs Incongruent__Y_: mean difference [95%CI] = -1.7 [-2.5 to -0.9], p<0.001). However the three conditions of incongruence did not statistically differ between each other (Incongruent__video_ vs Incongruent__XY_: mean difference [95%CI] = -0.6 [-1.2 to -0.2], p = 0.25; Incongruent__video_ vs Incongruent__Y_: mean difference [95%CI] = -0.5 [-1 to 0], p = 0.34; Incongruent__XY_ vs Incongruent__Y_: mean difference [95%CI] = 0.1 [-0.3 to 0.5], p = 0.94).

Medio-lateral drift: A significant main effect of Visual Feedback was found (F(3,57) = 4.1; p = 0.011, ɳ_p_ = 0.17). As shown on Fig 4C participants drifted in the medial axis in the Incongruent__video_ and Incongruent__XY_ conditions compared to the Congruent VF condition (Congruent vs. Incongruent_video: mean difference [95%CI] = -1.4 [-2.5 to -0.3], p = 0.011; Congruent vs. Incongruent__XY_: mean difference [95%CI] = -1.4 [-2.3 to -0.5], p = 0.019). No significant effect was observed between the Congruent VF condition and the Incongruent__Y_ condition (mean difference [95%CI] = -0.9 [-2 to 0.2], p = 0.059). However, the three conditions of incongruence did not differ between each other (Incongruent__video_ vs Incongruent__XY_: mean difference [95%CI] = 0.2 [-0.5 to 0.9], p = 0.89; Incongruent__video_ vs Incongruent__Y_: mean difference [95%CI] = 0.5 [-0.2 to 1.2], p = 0.59; Incongruent__XY_ vs Incongruent__Y_: mean difference [95%CI] = 0.2 [-0.4 to 0.8], p = 0.82).

Motor disturbances were observed in both medio-lateral (i.e. drift) and antero-posterior axis (i.e. reduced movement amplitude). In order to assess *a posteriori* whether both errors were related between each other, a Pearson coefficient correlation was performed on the mean of the three conditions of sensorimotor conflicts, revealing that errors in amplitude and drift during sensorimotor conflicts were not related (r = 0.26, p = 0.26).

### Objective 3—Test-retest reliability

As there was no statistical difference between the three conditions of incongruence on either sensory or motor disturbances, the Active_Incongruent__Video_, Active_Incongruent__XY_ and Active_Incongruent__Y_ conditions were averaged for each session. Results showed that test-retest reliability was good for both the sensory (ICC [95%CI] = 0.87 [0.72 to 0.95]) and motor (amplitude: ICC = 0.76 [0.50 to 0.90], drift: ICC = 0.76 [0.51 to 0.90]) disturbances.

## Discussion

The first objective of this study was to compare the effect of passive (visuo-proprioceptive conflict) and active (visuo-motor conflict) movement during incongruent and congruent visual feedback on sensory disturbances. We demonstrate for the first time that while sensory disturbances are evoked by both visuo-proprioceptive and visuo-motor conflicts, they were higher during visuo-motor than visuo-proprioceptive conflict.

It has been hypothesised that conflict-induced sensory disturbances are the result of a mismatch between the expected sensory consequence of movement (prediction based on the efference copy) and the actual sensory feedback [19,26,27]. During active movement, efference copy contributes to limb perception together with vision and proprioception [28,29], but this is not the case during passive movement. As a result, several studies show that perception of limb position is less accurate during passive than active movement [30–32]. Using mirror visual feedback and tendon vibration, previous studies showed even if proprioceptive and visual information are contradictory, they are combined together according to their respective reliability [33] to result in a unique percept of limb position and movement [34–36]. This contradictory information results in a movement illusion and bias in the sense of limb position [34–36], suggesting that our brain is easily tricked. However, we demonstrate that the conflict between vision and proprioception is detected and results in sensory disturbances (e.g. feelings of peculiarity, perception to having an extra-limb, losing control). Our results are in accordance with a previous study showing that incongruent proprioceptive inputs increase sensory disturbances in healthy participants [37]. Using extensor carpi radialis tendon vibration, the authors showed that sensations of swelling, foreignness and peculiarity were higher in participants who reported an illusion of wrist flexion than those who did not report any movement illusion during tendon vibration. The authors suggest that a conflict occurs between cortical representation of proprioceptive feedback, indicating a wrist flexion, and other proprioceptive inputs, for example sensory tactile receptors, indicating no movement [37]. Moreover, we demonstrate that the efference copy is a supplementary source of conflict between vision and proprioception and therefore reinforces the conflict, resulting in higher sensory disturbances in condition of active movement versus passive movements. It is important to note that the effect size was moderate (d = 0.41) and that the amplitude of the difference between active and passive movement during the Incongruent VF condition was small (inferior to the cut-off of 0.25), with a low level of certainty (as shown by the large confidence interval). In contrast, the effect size for the effect of Incongruent VF compared to the Congruent condition was large (ɳ_p_ = 0.53) in line with previous a previous study [4], with a high degree of certainty (as shown by the small confidence interval). Therefore, while the sensory disturbances evoked by the sensorimotor conflict are the results of both visuo-motor and visuo-proprioceptive conflicts, the role of the efferent copy appears to be limited.

The second objective of the study was to compare the effect of three conditions of incongruent visual feedback (relative to congruent feedback) on motor and sensory disturbances during active movements. Results show that all conditions of incongruence induced sensory disturbances and lowered movement amplitudes. Only the Incongruent__XY_ and Incongruent__Video_ conditions induced mediolateral drift compared to the Congruent VF. For the sensory disturbances, the effect size was large and the differences between the congruent and the incongruent conditions are important (superior to the cut-off of 0.25) with a high degree of certainty (small confidence intervals). For the motor disturbances, the effect sizes were moderate with a high degree of certainty (small confidence intervals). Contrary to what was expected, having visual feedback about movement errors does not impact on evoked disturbances. A previous study showed that viewing another person doing incongruent movement [38] induced motor disturbances, but this effect was not present when a robot arm was making incongruent movement. The authors suggest that viewing biological movement influences motor execution via activation of the mirror neurons system [38]. If this is applied to our study, then viewing a virtual upper limb moving incongruently with one’s own limb, no matter whether the movement is related or not to one’s own movements, could induce motor disturbances perhaps through mirror neuron system activation. This could explain why no difference was found between the three conditions of incongruence. In the studies assessing the effect of incongruent visual feedback on motor disturbances with the mirror in the frontal plane, authors showed that errors decrease with time [14,15]. In these experiments participants had access to visual feedback about movement errors and were able to do online corrections [14,15]. In our study, it was not possible to study the effect of time due to the small number of trials and their short duration. It could be hypothesised that having feedback on errors would lead to a decrease in motor disturbances over time.

The third objective was to test whether sensory and motor disturbances induced by sensorimotor conflicts were stable over time. Despite high variability between participants in our study and other studies [4,5,19], the test-retest reliability was very good for the sensory disturbances questionnaire and good for the motor disturbances measured by the exoskeleton, indicating a low variability within participants. Interestingly, both types of motor disturbances (reduction in amplitude and medio-lateral drift) were not related to each other, suggesting that different strategies are observed in response to the conflict, but that whatever the strategy is for a given individual, this is stable over time. Altogether, these results confirm that conflict-evoked disturbances are very different across participants, but reliable in a given participant.

Several limitations need to be highlighted. First, electromyography was not recorded to ensure that participants kept their arm muscles at rest during the passive condition. However, subjects were frequently reminded to keep their upper limbs as relax as possible. Secondly, the pattern of response was heterogeneous across all items. Indeed, participants mainly reported changes in feelings of peculiarity, in the impression of gaining a limb and the impression of losing control. Therefore, averaging all items led to low global scores. Thirdly, the level of certainty of the difference between visuo-proprioceptive and visuo-motor conflicts was low, and therefore this result needs to be replicated with a larger sample to confirm the involvement of the efference copy in sensory disturbances evoked by sensorimotor conflicts.

## Conclusions

The general aim of our study was to have a better understanding of the specific factors playing a role in conflict-induced disturbances. Our study has three major findings. Firstly, healthy participants report higher sensory disturbances during a visuo-motor conflict than a visuo-proprioceptive conflict. Our proposed explanation for this effect is that the conflict is more salient in the active (visuo-motor) than in the passive (visuo-proprioceptive) condition due to the production of an efference copy in the active condition. Secondly, viewing a virtual UL moving incongruently with our own movement induces motor disturbances, no matter whether the virtual upper limb is driven by our actual movement or not. Contrary to what was observed in the *sensory* disturbances (first objective) this result suggests slight involvement of the efference copy in *motor* disturbances. Conflict induced motor disturbances could be related more to the observation of another movement (perhaps through activation of the mirror neuron system).

Altogether, our results suggest that the generation of motor and sensory disturbances rely on different processes. Recently, we found that while sensory disturbances were increased by the presence of acute pain in healthy participants, it did not impact motor disturbances [4], supporting the idea of two different processes. However, as high variability was observed between participants in sensory and motor disturbances [4,5,19], independent effect on sensory and motor disturbances could be due to large intra-subject variability. In the third objective of this study, we demonstrate that the sensory disturbances questionnaire, amplitude and mediolateral drift are reliable outcomes to measure the disturbances induced by the conflict, despite the fact that high variability is observed between participants. Therefore, this methodological aspect reinforces the idea of two different processes underpinning sensory and motor disturbances.
